# Problem-Solving Skills Appraisal Mediates Hardiness and Suicidal Ideation among Malaysian Undergraduate Students

**DOI:** 10.1371/journal.pone.0122222

**Published:** 2015-04-01

**Authors:** Abbas Abdollahi, Mansor Abu Talib, Siti Nor Yaacob, Zanariah Ismail

**Affiliations:** 1 Faculty of Human Ecology, Universiti Putra Malaysia, Serdang, 43400, Selangor, Malaysia; 2 Family, Adolescent and Child Research Center of Excellent (FACE), Faculty of Human Ecology, Universiti Putra Malaysia, 43400, Selangor, Malaysia; Center for BrainHealth, University of Texas at Dallas, UNITED STATES

## Abstract

**Objectives:**

Recent evidence suggests that suicidal ideation is increased among university students, it is essential to increase our knowledge concerning the etiology of suicidal ideation among university students. This study was conducted to examine the relationships between problem-solving skills appraisal, hardiness, and suicidal ideation among university students. In addition, this study was conducted to examine problem-solving skills appraisal (including the three components of problem-solving confidence, approach-avoidance style, and personal control of emotion) as a potential mediator between hardiness and suicidal ideation.

**Methods:**

The participants consisted of 500 undergraduate students from Malaysian public universities.

**Results:**

Structural Equation Modelling (SEM) estimated that undergraduate students with lower hardiness, poor problem-solving confidence, external personal control of emotion, and avoiding style was associated with higher suicidal ideation. Problem-solving skills appraisal (including the three components of problem-solving confidence, approach-avoidance style, and personal control of emotion) partially mediated the relationship between hardiness and suicidal ideation.

**Conclusion:**

These findings underline the importance of studying mediating processes that explain how hardiness affects suicidal ideation.

## Introduction

Suicide is one of the serious and grave public health problems in many countries, and worldwide is the third highest cause of deaths among adolescents between 16 to 24 years old. Similarly, suicidal ideation is common during adolescence [1]. According to the World Health Organization, one million people have died from suicide, and one is dying by suicide every 40 seconds. It is also predicted that by 2020, one person will die every 20 seconds if urgent action is not taken [2]. Recent evidence suggests that suicidal ideation and attempts are increased in Asian countries among university students, indicating this group may be an at-risk population [3]. In Taiwan, around 7.5% of university students had ever planned to kill themselves [4], whereas 3.3% Korean university students had attempted suicide [5]. The situation of suicide among university students in Malaysia is no different from many other countries as well [6]. The National Health and Morbidity Survey reported that young Malaysians in the 16–24 year age group had the highest prevalence of acute and chronic suicidal ideation (10.0% and 26.0%, respectively), which may include a significant proportion of the Malaysian undergraduate students. The prevalence of suicidal ideation was higher in females and Indian race; however, gender and race differences in these relationships are unknown among Malaysian undergraduate students [7]. As university is a period of transition, major shifts may occur in social and psychological aspects of the individuals’ lives, and they may experience different levels of difficulties in academic pressures, occupational choices, and life goal decisions [3]. With regard to the above- mentioned reasons, suicidal ideation is on the increase among university students, making it important to identify early-warning signs to prevent suicide completion [3]. Therefore, this study was conducted to understand suicidal ideation and the variables associated with suicidal ideation among Malaysian undergraduate students.

Previous studies revealed that as problem-solving skills appraisal decreased (i.e., weaker belief in ability to solve problems, higher tendency to avoid rather than confront problems, and external control of emotion), suicidal ideation and depression considerably increased [8,9]. The term, “problem-solving skills appraisal,” is defined as one’s perception about one’s personal problem-solving style and the identification of one’s abilities and skills to solve problems [10]. The appraisal of problem-solving skills comprises three styles: problem-solving confidence, approach-avoidance style, and personal control of emotion. Problem-solving confidence is defined as having self-assurance in the face a wide range of problems and trusting in one’s own ability to solve the problems. The approach-avoidance style is defined as a tendency to approach or avoid facing problems. Personal control of emotion is defined as one’s ability to control his or her emotions and behavior while facing problems [11]. Several studies have shown that ineffective problem-solving abilities were associated with psychological stress[12,13], and suicidal ideation [14]. Conversely, effective problem-solving abilities were associated with effective emotional awareness [15], mental health, and life satisfaction [16].

Regardless of an abundance of study into the nature of the relationship between hardiness and problem-solving styles [12,13], no study, to our knowledge, has tested the relationships between problem-solving skills, hardiness, and suicidal ideation. Over the last three decades, there has been a great deal of research interest in the concept of “hardiness”. Hardiness has been developed into a theoretical framework known as the hardiness construct [17], which examines the reasons why some individuals, even under stressful conditions, are able to deal with problems, and why some individuals in non-stressful conditions are not able to deal with problems. The term hardiness was introduced by Kobasa [18] as a way of understanding a person’s relation with others, his or her goals and problems. Kobasa, Maddi, and Kahn [17] defined hardiness as an ability incorporating three components—commitment, control, challenge—to prepare an individual to handle problematical life events [19]. Commitment is defined as a person committed to activities. Each activity is meaningful and interesting for him/her. Control is defined as a person believing that he/she can control or influence their life experiences. Challenge is defined as a person perceiving the world as an opportunity to develop, as well as being a good learner [17]. Research evidence has supported Kobasa's argument that hardiness plays an influencing role against suicidal ideation [20,21]

According to Kobasa’s definition, two mechanisms have been proposed to explain the effect of hardiness: hardy individuals have the motivation to carry on effective problem-solving skills during stressful situations [22], and appraise stressful conditions as being more challenging and controllable and less threatening. Additionally, hardy individuals seek to gain experience from stressful conditions [23]. Garrosa et al. [22] concluded that individuals with high levels of hardiness applied effective managerial strategies to reduce stress and hopelessness. They are more flexible in facing problems [24], and prefer to use rational-oriented coping styles rather than emotional-oriented coping styles in managing stressful conditions [23]. Studies have reported that individuals who are high in hardiness are more likely to report happiness, life satisfaction, and mental and physical health [23,25,26], while other studies have reported that individuals who are low in hardiness are more likely to report mental disorders, such as depression, anxiety, and stress [27]. The present study is designed to understand undergraduate students’ hardiness in relation to their suicidal ideation.

There is no academic literature concerning the relationships between problem-solving skills appraisal, hardiness, and suicidal ideation, because, thus far, many of the studies on hardiness have focused on coping with stress [28,29]. This study aims to expand the hardiness theory on suicidal ideation in undergraduate students. Our literature review highlights the lack of research about suicidal ideation and hardiness, which is considered to be vital for the improvement of public health. There is still a gap in the association between hardiness and suicidal ideation. The reasons why individuals with lower hardiness experience high levels of suicidal ideation is ambiguous, because most of the studies in this area only addressed the association between hardiness and suicidal ideation [30], without considering other variables influencing the association. Therefore, this study can be useful in clarifying the association between hardiness and suicidal ideation and the variable influencing the association. Based on the hardiness theory [31], it is conceivable that poor hardiness prevents individuals from engaging in coping responses to stress and resistance to stressful situations during times of stress [28,29], and may lead to lower levels of problem-solving skills and higher levels of suicidal ideation.

### Hypotheses

The current study sought to examine a number of hypotheses between variables and suicidal ideation. Firstly, a positive relationship exists between poor problem-solving skills appraisal (including the three components of problem-solving confidence, approach-avoidance style, and personal control of emotion) and suicidal ideation. Secondly, a negative relationship exists between hardiness and suicidal ideation. Thirdly, problem-solving skills appraisal (including the three components of problem-solving confidence, approach-avoidance style, and personal control of emotion) mediates the relationship between hardiness and suicidal ideation. Lastly, gender and race moderate the relationship between exogenous variables, such as problem-solving confidence, approach-avoidance style, personal control of emotion, hardiness, and suicidal ideation as an endogenous variable.

## Methods

### Participants

A total of 500 undergraduate students from two Malaysian public universities participated in this study (male = 46%, n = 230, and Female = 54%, n = 270, aged from 18 to 24 years, Mean = 20.28, SD = 2.58). The ethnicity of the participants included Malay 47.8% (n = 223), Chinese 26.8% (n = 134), Indian 22.6% (n = 113), and others 2.8% (n = 14). In terms of marital status, 76.4% (n = 382) of participants were single, 22.6% (n = 113) of participants were married, and 1% (n = 5) of participants were separated or widowed. Of the 500 participants, 30% (n = 150) were in the freshman year; 23% (n = 115) were in the sophomore year; 25% (n = 125) were in the junior year, and 22% (n = 110) were in the senior year. In terms of religion, 47.2% (n = 224) of the participants were Muslim, 26.2% (n = 131) Hindus, 21.2% (n = 106) Buddhists, 3% (n = 15) Taoists, and 0.8% (n = 4) were of other religious affiliations.

### Procedures

The Malaysian Ministry of Science, obtained permission for gathering data from two public universities. The faculty members were categorized into three fields (science, social science, and technical). Then, a faculty was chosen randomly from each field. After that, four classes (first, second, third, and last year undergraduate students) from each faculty were randomly selected, and data were collected during one of the regularly scheduled classes. The inclusion criteria for the participants were that (a) they should be university students and (b) be between the ages of 16 and 24 years old. The packages of questionnaires were distributed among undergraduate students. Each package contained an introductory letter about the aims of the study and four questionnaires (one of them was a demographic questionnaire). A total of 550 questionnaires were distributed among undergraduate students, of which 500 (90%) usable questionnaires were returned; 6% (33) refused to complete the questionnaires.

### Ethical statement

The ethics committee of Universiti Putra Malaysia (UPM/TNCPI/1.418.1) approved the study. Written informed consent was obtained from the participants in this study for their involvement in the research. This included the purpose of the research and the fact that their participation was voluntary. The consent procedure was approved by the ethics committee and Malaysian Ministry of Science.

### Measures

#### Beck scale for suicidal ideation [32]

The Beck Scale for suicidal ideation is a self-report measure with 21 items that assess suicidal ideation, planning and suicidal intent in the past week. A 3-point Likert scale from 0 to 2 was used for all questions, and the range of scores is from 0 to 38. If a participant had a high score in BSSI it meant that he/she has a higher suicide risk and vice versa, and that he/she has passive or active thoughts about killing himself/herself. Previous studies showed that BSSI had an acceptable internal consistency and convergent validity [33,34]. The current study showed a good convergent validity (AVE: 0.69), and the construct reliability (CR: 0.81) for this instrument.

#### Personal views survey, third edition revised [35]

The revised Personal Views Survey Third Edition is comprised of 18 items that assess the attitudes of commitment, control, and challenge [35]. The hardiness literature does not support the separation into subscales of the hardiness components of commitment, control, and challenge [36]. Therefore, in this study, the sum of three scores was used. The scores range from 0 to 54. A 4-point Likert scale was used for all questions ranging from 0 (not at all true), 1 (somewhat true), 2 (true), and 3 (very true). Studies have shown an acceptable internal consistency (test-retest coefficient, interval between 2 and 4 weeks): for commitment (α: 0.70–0.75); for control of emotion (α:0.61–0.84); for challenge (α:0.60–0.71); and for total hardiness(α:0.80–0.88) [35]. In addition, the construct validity of PVS III-R reported α: 0.70 to 0.84 [37]. The construct validity of the challenge was (α: 0.62), commitment (α: 0.59), and control was (α: 0.46) [38]. The present study showed a good convergent validity (AVE: 0.60), and the construct reliability (CR: 0.75) for this instrument.

#### Problem-solving inventory [39]

The problem-solving inventory comprises 32 items that measure the perceptions of one’s problem solving beliefs and style in facing problems and difficulties in one’s daily life [39]. All questions were based on a 6-point Likert scale from 1 (strongly agree) to 6 (strongly disagree). This questionnaire consists of three factors: (a) Problem-solving confidence (PSC), (b) Approach-Avoidance Style; (c) Personal control of emotion. The problem-solving inventory provides scores on three problem-solving dimensions [39]; therefore, in this study, three factors were evaluated separately. Based on a wide range of studies, this questionnaire showed good validity [39,40]. The present study showed a good convergent validity for PSC, AAS, and PCS with (AVE: 0.58, 0.53, and 0.51 respectively), and the construct reliability (CR: 0.77, 0.74, and 0.71, respectively).

#### Demographics

A self- report questionnaire was provided to obtain demographic information, such as gender, age, race, educational levels, religious affiliation, and marital status.

### Pilot study

A pilot study was conducted on sixty undergraduate students to determine the reliability of the tools. The Cronbach’s Alpha coefficients for the questionnaires in the pilot study were as follows: (1) Beck Suicidal Ideation Scale (α: 0.73); (2) Personal Views Survey, Third Edition Revised (α: 0.78); and (3) Problem-Solving Inventory (α: 0.79). In general, the respondents of the pilot study gave positive feedback toward the general structure and presentation of the questionnaire. To improve the face validity of the scales, the survey questionnaire was further refined based on some comments collected from the participants. In order to assess the face validity and content validity, and to ensure its adaptability to the local cultural context, the instrument was reviewed and approved by the Faculty of Human Ecology, Universiti Putra Malaysia.

### Analysis

We employed Structural Equation Modelling (SEM) analysis. According to Kline [41], the advantages of employing SEM are that it (a) improves statistical estimation by taking into account the measurement error in the estimation process, (b) enables the testing of multiple relationships simultaneously, (c) tests much more complex models, such as testing mediation, moderation, and provides goodness of fit indices for the model tested, and (d) provides better identification for convergent validity, construct reliability, and discriminant validity for the instruments.

The Average Variance Extracted (AVE), and Construct Reliability (CR) were performed for measuring the convergent validity and construct reliability of the instruments. Convergent Validity refers to a set of indicators (items) that presume to measure a construct and it should be above the critical value of 0.5 [42]. The construct reliability is comparable to Cronbach alpha value, but it is not Cronbach alpha and it should be above the critical value of 0.7 [42]. Discriminant validity refers to whether a construct is truly distinct from another construct [42]. In order to show the discriminant validity the average variance extracted for two constructs should be bigger than the square of the correlation between the two constructs [42]. The results showed that the constructs had suitable discriminant validity.

### Data preparation

The missing range of items and parcels were from 1.81% to 2.81%; therefore, for addressing missing data a multiple imputation method in AMOS 20 software was applied. Outliers of 4% (n = 22) were excluded from the analyses (those scoring three standard deviations from the mean). The data were distributed normally, because the skewness values were from -1.77 to 1.68, and the kurtosis values were from -2.61 to 2.11 for all variables. Byrne [43] stated that if the skewness value is between -2 to +2, and the kurtosis value is between -7 to +7; multivariate normality of the data could be assumed. For model fit, Kline [44] suggested using model fit indexes, including the chi square/degree of freedom ratio (CMIN/DF), the comparative-fit index (CFI), the goodness-of-fit index (GFI), and the Tucker-Lewis Index (TLI). A rule of thumb for the fit indices is that values equal or greater than 0.90 indicate acceptable fit [44]. Furthermore, the model may be classified as acceptable if the root mean squared error of approximation (RMSEA) is between 0.03 and 0.08. AMOS 20 software was utilized for analysing the data.

## Results

### Descriptive statistics

Table 1 reports the inter-correlations between hardiness, suicidal ideation, problem-solving confidence, approach-avoidance style, and personal control of emotion for male and female students, as well as the standard deviations, actual range and the means (Table 1).

**Table 1:** Correlation between study variables for male and female students, and the mean, SD and actual range.

Variables	1	2	3	4	5
(1) Hardiness	1	-.433**(-.354**)	-.283**(-.217**)	-.300*(-.150*)	-.259*(-.129*)
(2) SI^1^		1	.388**(.488**)	.252*(.488**)	.359**(.415*)
(3) PSC^2^			1	.420***(.367***)	.569** (.401**)
(4) AAS^3^				1	.464*** (.517***)
(5) PC^4^					1
Mean	31.21(30.28)	14.88(16.24)	28.41(27.21)	46.88(47.81)	19.78(20.12)
SD	12.78(11.87)	7.89(6.32)	9.58(8.68)	12.11(11.12)	5.18(6.11)
Actual range	14-53(13–52)	0-33(0–33)	16-53(16–53)	19–71 (19–70)	14-27(14–27)

Note:

*P<.05

**P<. 01

***P <. 001

1: Suicidal Ideation,

2: Problem-solving Confidence,

3: Approach-Avoidance Style,

4: Personal Control. Results for male are presented first, and results for female are presented in a parenthesis.

### Measurement model

This model included problem-solving skills appraisal (including the three components of problem-solving confidence, approach-avoidance style, and personal control of emotion), hardiness, and suicidal ideation as the latent variables depicted good fit indices: CMIN/DF = 2.95, *p*<. 01, CFI = 0.953, GFI = 0.903, TLI = 0.940, RMSEA = 0.063. According to Kline [44], the model provides an acceptable fit for the model.

### Structural model

**Fig 1:**
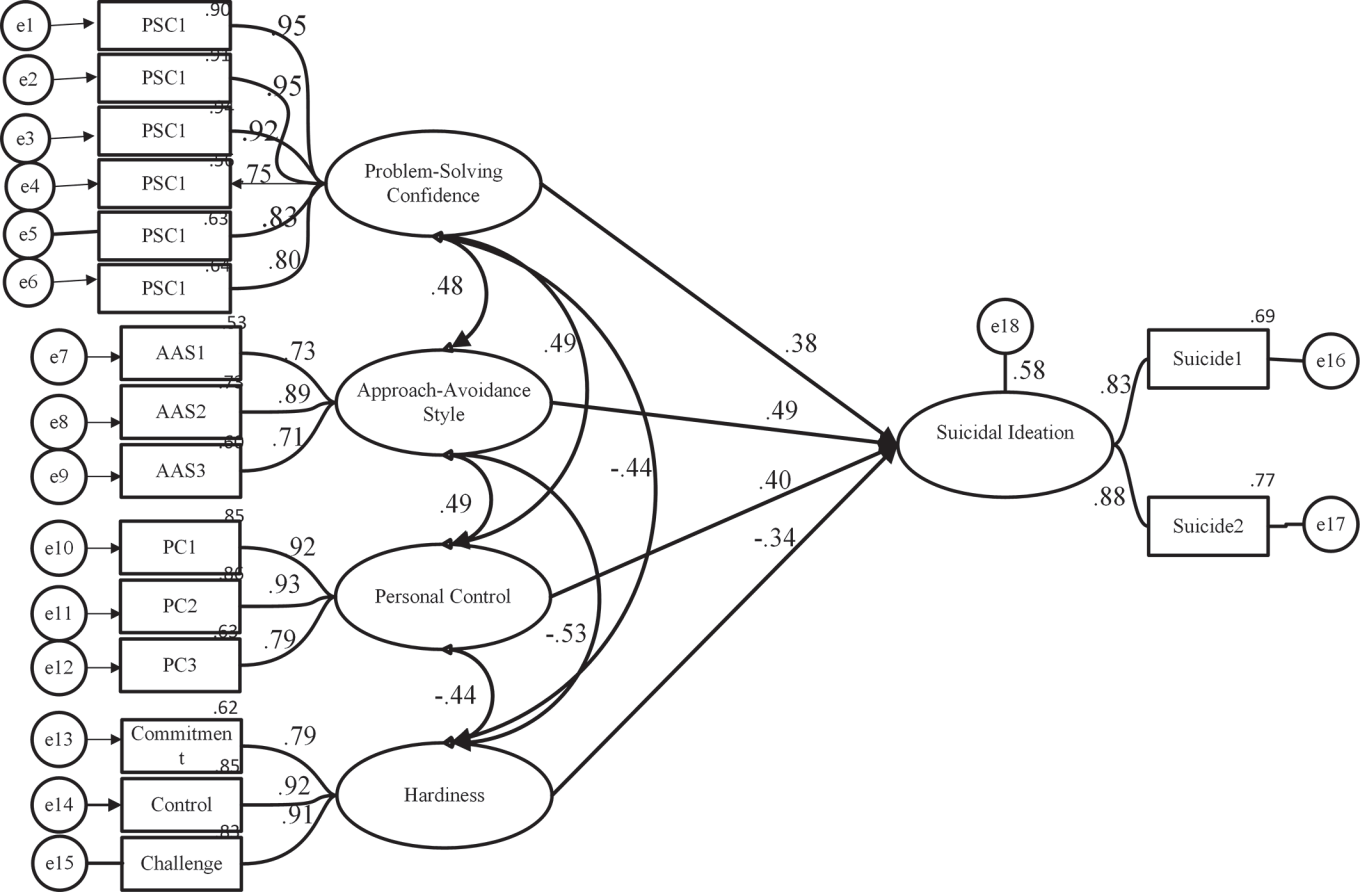
Structural model of suicidal ideation. For all estimate *p*<0.01. Exogenous Variables including (Problem-solving Confidence, Approach-Avoidance Style, Personal Control, and Hardiness).

The model included problem-solving skills appraisal (including the three components of problem-solving confidence, approach-avoidance style, and personal control of emotion) and hardiness as exogenous variables; suicidal ideation was an endogenous variable. As it can be seen from the Fig 1, problem-solving confidence, approach-avoidance style, personal control of emotion, and hardiness had significant relationships with suicidal ideation. It can be seen from the data in Fig 1 that ineffective problem-solving confidence, avoiding style and external personal control of emotion were positively associated with suicidal ideation, and a negative association existed between hardiness and suicidal ideation in undergraduate students. The findings of this study demonstrated that problem-solving confidence, approach-avoidance style, personal control of emotion, and hardiness are valuable predictors of suicidal ideation. These variables explained 58.0% of the variance in suicidal ideation.

### Mediation test of problem-solving skills appraisal

The model included hardiness as an exogenous variable; and problem-solving confidence, approach-avoidance style, personal control of emotion, and suicidal ideation as endogenous variables. Hair, Black, Babin, Anderson, and Tatham [42] indicated that, “a mediation effect is created when a third variable/construct intervenes between two other related constructs” (p. 866). The multi-model analysis was used for testing mediation effects [45]. Hair et al. [42] indicated that: if the relationship between the exogenous variable and the endogenous variable is zero when the mediator is included, full mediation is established. However, if the relationship between the exogenous variable and the endogenous variable is reduced when the mediator is included, partial mediation is established.

In order to establish a mediation effect in the overall model, it must be shown that full mediation model is better than an indirect model [45]. The goodness-of-fit indices for full mediation and the indirect model could be compared based on nested model comparisons [44]. The full mediation model showed (Chi-square: 2.362. *p*>0.01, GFI: 0.901, CFI: 0.901, TLI: 0.899, and RMSEA: 0.052), and the indirect model showed (Chi-square: 2.421, *p*> 0.01, GFI: 0.796, CFI: 0.889, TLI: 0.901, and RMSEA: 0.056). Although both models fit the data according to the nested model comparison, the full mediation model showed a significantly better fit than the indirect model, because the full mediation model showed smaller AIC (Akaike Information Correction: 3987.881), to compare the indirect model (Akaike Information Correction: 4080.969), and the PNFI (Parsimony Normed Fit Index) of the full mediation model was (0.785) bigger than the PNFI of the indirect model (0.781).

According to the above-mentioned condition indicated by Hair et al. [42], problem-solving confidence, approach-avoidance style, and personal control of emotion mediate the relationship between hardiness and suicidal ideation. As demonstrated in Table 2, the results show that in the direct model the relationship between hardiness and suicidal ideation was significant (β = -0.443). In addition, the results based on the mediation model show significant relationships between problem-solving confidence, approach-avoidance style, and personal control of emotion with hardiness (β = -0.504, -0.437, and -0.666 respectively), and hardiness with suicidal ideation (β = -0.261). However, although the size of the standard regression weight for the direct relationship between hardiness and suicidal ideation reduced when problem-solving confidence, approach-avoidance style, and personal control of emotion were added as mediating variables in the mediation model, it was still significant. Therefore, the partial mediation of problem-solving skills appraisal (problem-solving confidence, approach-avoidance style, and personal control of emotion) on the relationship between hardiness and suicidal ideation was supported.

**Table 2:** Standard Regression Weight in the Full Mediation, direct, and indirect Model.

DV		IV	Mediation Model	Direct Model	Indirect Model
PSC^1^	<—-	Hardiness	-.504**		-.518***
AAS^2^	<—-	Hardiness	-.437**		-.455***
PC^3^	<—-	Hardiness	-.666**		-.678***
Suicidal Ideation	<—-	PSC^1^	.181***		.323**
Suicidal Ideation	<—-	AAS^2^	.268***		.347***
Suicidal Ideation	<—-	PC^3^	.361***		.564***
Suicidal Ideation	<—-	Hardiness	-.261***	-.443***	

Note:

**P<. 01

***P <. 001

1: Problem-Solving Confidence,

2: Approach-Avoidance Style,

3: Personal Control.

### Moderation test of gender

A comparison between “the variant group model” and “the invariant group model” showed that the variant group model with (CMIN: 918.898, df = 400, *p* < 0.01, RMSEA = 0.052, CFI = 0.929, GFI = 0.891, NFI = 0.901), and the invariant group model with (CMIN: 1276.449, df = 476, *p* < 0.01, RMSEA = 0.058, CFI = 0.913, GFI = 0.823, NFI = 0.818) were significant; however, the variant group model was better than the invariant group model, because the chi-square was smaller [42,46]. According to the invariant group model (CMIN: 1276.449, df = 476, and *p*< 0.01) in “assuming that the unconstrained model is correct," the findings showed that the impact of likely differences across gender was significant.

The results indicated that the relationships between problem-solving confidence and approach-avoidance style with suicidal ideation for both male and female students were significant (See Table 3), while there were differences in the value of standard regression weight for male and female students (See Table 3). Therefore, the moderating effects of gender on these paths were not supported. In addition, the results revealed that there was no significant relationship between hardiness and suicidal ideation for female students (β = .-0.272), while the path hypothesis for male students was significant (β = -0.313) (Table 3). Therefore, the moderating effect of gender on the path relationship between hardiness and suicidal ideation was supported. Our finding supports a previous study representing that male students scored higher on hardiness [47]. Moreover, the results revealed that there was a significant relationship between personal control of emotion and suicidal ideation for both groups (Table 3). Therefore, the moderating effect of gender on the path relationship between personal control of emotion and suicidal ideation was not supported.

**Table 3:** Standardized Regression Weights (Gender variant Model).

Hypothesis			S.E.	C.R.	Standard Estimate
SI^4^	<—-	PSC^1^	1.412(1.388)	1.045(1.499)	.488**(.588**)
SI^4^	<—-	AAS^2^	.888(1.174)	.611(1.012)	.252*(.488**)
SI^4^	<—-	Hardiness	.084(.215)	-7.434(-5.569)	-.313** (-.274)
SI^4^	<—-	PC^3^	.128(.232)	.301(.348)	.159*(.215**)

Note:

*P<. 05

**P<. 01

1: Problem-Solving Confidence,

2: Approach-Avoidance Style,

3: Personal Control,

4: Suicidal Ideation. Results for male are presented first, and results for female are presented in a parenthesis.

### Moderation test of race

As depicted in Table 4, the moderating effect of race on the path relationship between problem-solving confidence and suicidal ideation was supported. While there were differences in the value of the standard regression weight between hardiness and suicidal ideation, this path was significant for the Malay, Chinese, and Indian groups (See Table 4). Therefore, the moderating effect of race on this path was not supported. In addition, the results indicated that the relationship between approach-avoidance style and personal control of emotion with suicidal ideation for Malay, Chinese, and Indian groups were not significant. Therefore, the moderating effects of race on these paths were not supported.

**Table 4:** Standardized Regression Weights (Race Variant Model).

Hypothesis			Malay	Chinese	Indian
SI^1^	<—-	PSC^2^	.412**	.373**	.112
SI^1^	<—-	AAS^3^	.314	.412	.236
SI^1^	<—-	Hardiness	-.454***	.303***	.294***
SI^1^	<—-	PC^4^	.168	.139	.169

Note:

**P<. 01

***P <. 001

1: Suicidal Ideation,

2: Problem-solving Confidence,

3: Approach-Avoidance Style,

4: Personal Control.

## Discussion

In particular, our findings demonstrated that lower hardiness and poor problem-solving confidence, avoiding style, external control of emotion significantly predicted suicidal ideation among undergraduate students.

The empirical findings showed that problem-solving confidence, approach-avoidance style, and personal control of emotion partially mediated the relationship between hardiness and suicidal ideation. That is, hardy individuals were less likely to experience suicidal ideation. High-hardy individuals engage in more effective problem-solving skills appraisal and less ineffective problem-solving skills appraisal than do low-hardy individuals. Therefore, if hardiness and problem-solving skills appraisal are increased, suicidal ideation will decrease. Recently, interest into the concepts of resilience in the face of suicidal ideation has increased, but most of the studies only examined resilience factors as linear relationship with suicidal ideation [48], and problem-solving skills appraisal has not been studied as a mediator for these variables. Therefore, hardiness and effective problem-solving skills appraisal need to be understood as influencing factors that can decrease the likelihood of suicidal ideation. By finding a relationship between hardiness and problem-solving skills appraisal for the undergraduate students' sample, the present findings confirmed the previous results and improved them in three main ways.

First, the present findings showed that hardiness may constitute a protective factor for undergraduate students’ sample, especially for individuals with suicidal ideation [24]. The study identified several risk factors for suicidal ideation in individuals, and these factors increased the likelihood of suicidal ideation [8]. Poor problem-solving skill is one of the psychological risk factors for suicidal ideation [49]. Although an understanding of suicidal factors can improve the prediction of suicidality and develop clinical interventions, it is still not comprehensive. The results of this study indicated that hardiness acts as protective factor and that when studied in relationship with risk, it can enhance predictive validity. Furthermore, it can be helpful in identifying individuals who are at risk of suicide, so that interventions may be implemented to reduce suicidal thinking and the risk of suicide.

Second, the current study examined the interaction between hardiness and problem-solving styles. Formerly, hardiness was studied in relation to stressful life events [50], but stress may not be an exact predictor of suicidality among individuals with mental disorder [51]. This study focused on one of the risk factors of suicidal ideation, namely, ineffective problem-solving skills. The finding that hardiness is a significant factor against ineffective problem-solving skills indicated that the effect of hardiness is not limited to stressful life events and suggests that hardiness may be a significant protective factor for suicidality. Although further investigation is needed, the present study suggests that hardiness plays an important role against suicidal ideation.

Third, the current study used the hardiness theory, which indicated that a positive appraisal of events may provide a source of resilience. The findings showed that hardiness could be useful and protective against ineffective problem-solving styles and suicidal ideation among Malaysian undergraduate students. Hardy individuals who tend to believe that the outcomes are promising and positive, [52] may provide a sense of resilience during difficult situations, and are less likely to experience suicidal ideation [30].

### Implications for practice

There are two significant implications for clinical treatment in the present study. First, when assessing the risk of suicide in individuals, it is important to account for the presence of hardiness in addition to risk factors, such as ineffective problem-solving skills. Previous studies indicated hardiness is an ability that prepares an individual to cope with problems [18,53]. Hardy individuals have a more optimistic viewpoint than individuals who are low in hardiness. They are more flexible in facing problems [24], and prefer to use rational-oriented coping styles rather than emotional-oriented coping styles in managing stressful conditions [23]. Second, hardiness training may help individuals to engage in effective problem-solving skills and decreases the amount of suicidal ideation, and it may be a significant factor to incorporate into suicide treatment programs. Reducing suicidal risk in an individual is a main focus of any clinical intervention.

### Strengths and limitations

The primary strength of this study is the role of problem-solving skills appraisal as a mediator between hardiness and suicidal ideation among undergraduate students. The results highlight the role of hardiness in lowering ineffective problem-solving skills and suicidal ideation in undergraduate students. One important limitation of this study is its reliance on self-report questionnaires. Although the measures used in the study are psychometrically adequate, a multi-method approach would be superior and lend incremental validity to the current study. Studies have shown that when participants completed questionnaires of mental health and hardiness simultaneously, they may tend to overlap perceptions of mental health with well-being. Consequently, it is plausible that clinical interviews would enable us to overcome the aforementioned limitation. In addition, this study employed a cross-sectional design and does not allow for causal interpretation about relationships between variables. Lastly, future studies could investigate the role of hardiness in the process of suicidal individuals' treatment.

In conclusion, the present study showed that hardiness decreased the risk of suicidal ideation by mediating effective problem-solving skills among undergraduate students.
